# Enormously large tippers observed in southwest China: can realistic 3-D EM modeling reproduce them?

**DOI:** 10.1186/s40623-023-01863-y

**Published:** 2023-07-14

**Authors:** Shan Xu, Chaojian Chen, Mikhail Kruglyakov, Alexey Kuvshinov, Rafael Rigaud, Xiangyun Hu

**Affiliations:** 1 School of Geophysics and Geomatics, China University of Geosciences, Wuhan, China; 2 Institute of Geophysics, ETH Zürich, Zürich, Switzerland; 3 Department of Earth and Environmental Sciences, Ludwig Maximilian University of Munich, Munich, Germany; 4 Department of Physics, University of Otago, Dunedin, New Zealand; 5 State Key Laboratory of Geodesy and Earth’s Dynamics, Innovation Academy for Precision Measurement Science and Technology, CAS, Wuhan, China; 6 Institute of Solar-Terrestrial Physics, Siberian Branch of Russian Academy of Sciences, Irkutsk, Russia

**Keywords:** Geomagnetic observatory data, 3-D electromagnetic modeling, Tippers

## Abstract

**Abstract:**

Vertical magnetic transfer functions (tippers) estimated at a global/continental net of geomagnetic observatories/sites can be used to image the electrical conductivity structure of the Earth’s crust and upper mantle (down to around 200 km). We estimated tippers at 54 geomagnetic observatories across China, aiming eventually to invert them in terms of subsurface three-dimensional (3-D) conductivity distribution. Strikingly, we obtained enormously large tippers at three inland observatories in southwest China. Large tippers are often observed at coastal/island observatories due to high conductivity contrasts between resistive bedrock and conductive seawater. However, tippers at those inland observatories appeared to be a few times larger than coastal/island tippers. As far as we know, such large tippers (reaching value 3) were never reported in any region worldwide. We perform electromagnetic simulations in 3-D conductivity models mimicking the geological setting and demonstrate that enormously large tippers are feasible and can be attributed to a current channeling effect.

**Graphical Abstract:**

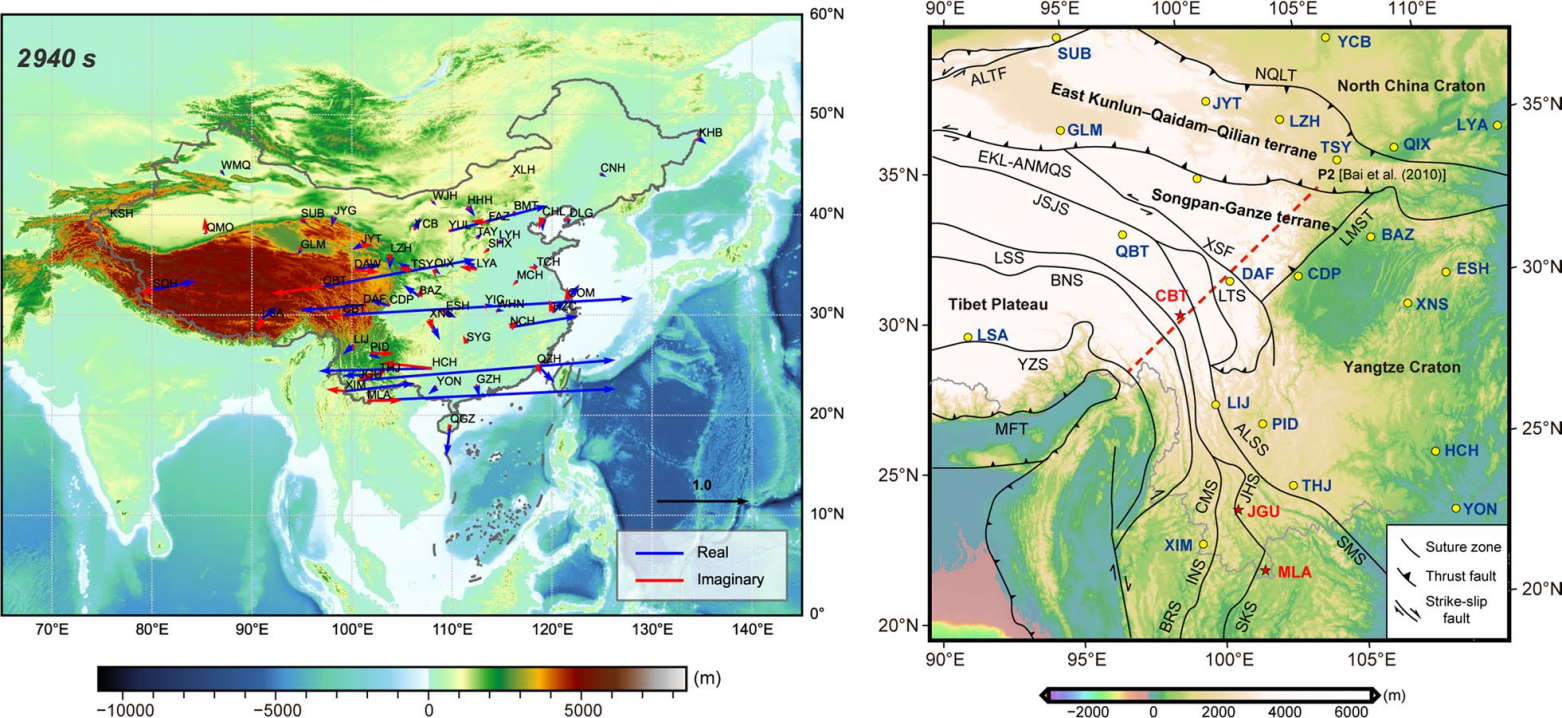

## Introduction

One of the physical parameters describing Earth’s interior—electrical conductivity—being sensitive to temperature and the presence of conductive phases such as water and melts (Karato and Wang 2013; Yoshino and Katsura 2013) serves as a valuable source of information on the thermal and compositional state of our planet, thus helping to understand its past evolution and modern dynamics.

As well known, magnetic field variations with periods less than a few hours can be used to constrain conductivity distribution at depths down to $\sim$ 200–300 km. The source responsible for these variations is conventionally approximated by a vertically incident plane wave of variable polarization. Such approximation allows researchers to introduce the so-called vertical magnetic transfer function, tipper, which relates in the frequency domain and at a given location the vertical component of the magnetic field to its horizontal components (Berdichevsky and Dmitriev 2008). Estimated at a grid of observations, tippers can be further inverted in terms of three-dimensional (3-D) conductivity distribution at crustal and upper-mantle depths. One of the prerequisites to obtaining trustworthy 3-D conductivity model(s) in the region of interest is performing magnetic field measurements at a grid that is as regular as feasible. Moreover, constraining conductivity at the maximum possible depths, the measurements should last as long as required to obtain reliable tippers at the longest period (about 3 h) where the tipper concept works.

Wang et al. (2014) performed the first-ever continental-scale 3-D inversion of tippers, which were estimated from a unique data set—minute-mean time series of the magnetic field acquired at 57 sites across Australia during 1989–1990 years in the framework of the Australian Wide Array of Geomagnetic Stations project (Chamalaun and Barton 1993). As a result, Wang et al. (2014) obtained a 3-D conductivity model of the Australian continent, which revealed various crustal and upper-mantle conductivity structures corresponding well with the region’s tectonics and with velocity anomalies evident in seismic tomography models of Australia and its surroundings.

**Fig. 1:**
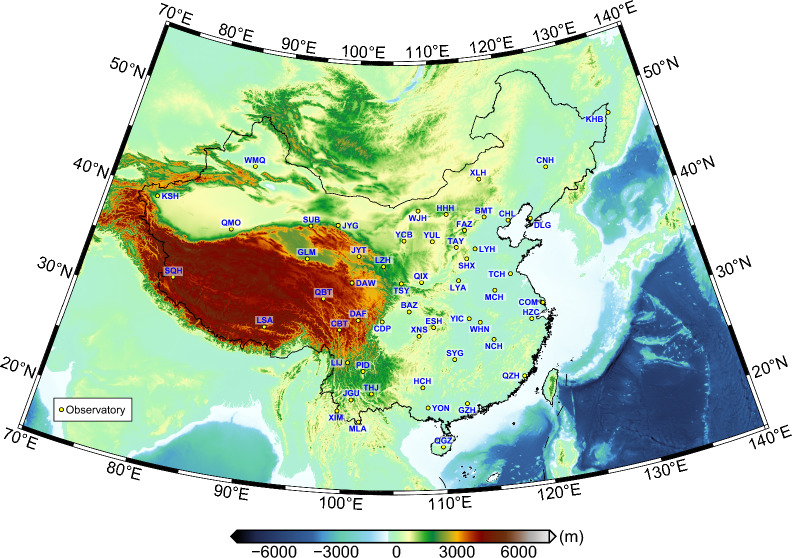
Topography/bathymetry of the region and distribution of geomagnetic observatories used in this study

The success of their study inspired us to pursue tippers’ estimation from the data continuously collected at 54 geomagnetic observatories across China, aiming eventually their 3-D inversion to image the regional 3-D conductivity structure beneath the Chinese mainland. Figure 1 shows the distribution of the considered observatories.

Estimating tippers from these data revealed their enormously large values at three inland observatories in southwest China. It is well known that in the considered period range between 5 min and 3 h, large tippers (typically, having magnitudes < 1.5) are mainly observed at the island and coastal sites due to the high lateral conductivity contrast between resistive continental bedrock and conductive seawater (e.g., Thiel et al. 2009; Rigaud et al. 2021; Kruglyakov and Kuvshinov 2022; Chen et al. 2023). However, tippers at *inland* observatories, abbreviated as CBT, JGU, and MLA in Fig. 1, appeared to be at least twice as large as the largest observed island/coastal tippers. To our knowledge, such large tippers (reaching value 3) were never reported in the literature.

In this study, we perform a comprehensive model study and suggest a 3-D model of the region that explains exceptionally large inland tippers.

## Tippers and their estimation

As stated in the Introduction, tipper relates the vertical magnetic field, $B_z$, with the horizontal magnetic field, ${\textbf{B}}_\tau = (B_x, B_y)$, as

1 $$\begin{aligned} B_z({\textbf{r}},\omega )=T_{zx}({\textbf{r}},\omega )B_x({\textbf{r}},\omega ) + T_{zy}({\textbf{r}},\omega )B_y({\textbf{r}},\omega ), \end{aligned}$$

where ${\textbf{r}}$ is the coordinates of the observation site, $\omega = \frac{2\pi }{T}$ is the angular frequency, and *T* is the period of variations. In our implementation, $x-$ and $y-$ and $z-$axes are directed to the geographic north, east, and down, respectively. Due to the assumption that the vertically incident plane wave approximates the source, the vertical magnetic field, and thus tippers, are zero above the Earth’s models where the conductivity varies only with depth. Consequently, non-zero tippers are indicators of lateral conductivity gradients in the Earth’s subsurface. Tippers are complex-valued quantities and are often displayed as (induction) arrows (separately for real and imaginary parts). In the Parkinson’s convention (Parkinson and Jones 1979), which we use in our study, the real-part arrows point towards anomalous current concentration. The length of the arrows is proportional to the intensity of the anomalous currents. One can interpret tippers as a measure of the tipping of the magnetic field out of the horizontal plane in the vicinity of 2-D and/or 3-D conductivity structures. As shown by Marcuello et al. (2005), one helpful plausibility check for the observed (i.e., estimated from the data) or/and modeled tippers is as follows: at periods where the real parts of tippers reach a maximum (or a minimum) value, the corresponding imaginary parts change the sign, meaning that the imaginary parts can be considered as derivatives (with respect to period) of the corresponding real parts.

To estimate tippers, we used observatory minute-mean time series of the magnetic field for the 2008–2019 period. Long time series allowed us to find time intervals (usually of one-month length) at which the tipper estimates were the most credible, i.e., demonstrated high squared coherency (to be discussed later), smooth behavior with respect to the period, and small uncertainties.

Most data are from observatories run by the Chinese Earthquake Administration, except those from BMT, GZH, KHB, and LZH observatories that are retrieved from the British Geological Survey repository (Macmillan and Olsen 2013). The data were first corrected for jumps, drifts and outliers. Tippers were estimated at 16 periods between 300 and 10000 s. To estimate tippers at each period, *T*, we split the data of length *L* (as mentioned above, usually taken as one month) into overlapped (50 %) tapered (using Hanning window) segments of three-period length. Data in these segments were Fourier transformed, giving $N=2L/3T-1$ estimates of the corresponding component spectrum. Tippers and their uncertainties were then calculated using a robust linear regression approach based on the Huber norm (Püthe and Kuvshinov 2014).

The quality of the tipper’s estimates at a given period is usually evaluated by squared coherency $R^2({\textbf{r}},\omega )$, a number showing, in our case, the correlation between input, ${\textbf{B}}_\tau$, and output $B_z$ signals at location ${\textbf{r}}$. If $R^2=1$, all the variability in the output signal is explained by the variability in the input signals indicating a perfect correlation (and thus the excellent quality of the estimated tippers). On the other hand, $R^2 = 0$ means no correlation between ${\textbf{B}}_\tau$ and $B_z$; hence all variability is due to random fluctuations or/and the source spatial structure incompatible with a plane wave. Tippers’ estimates obtained at observatories used in this study mostly have relatively high $R^2$ ($>0.6$), indicating their good quality.

## Detecting enormously large tippers in southwest China

**Fig. 2:**
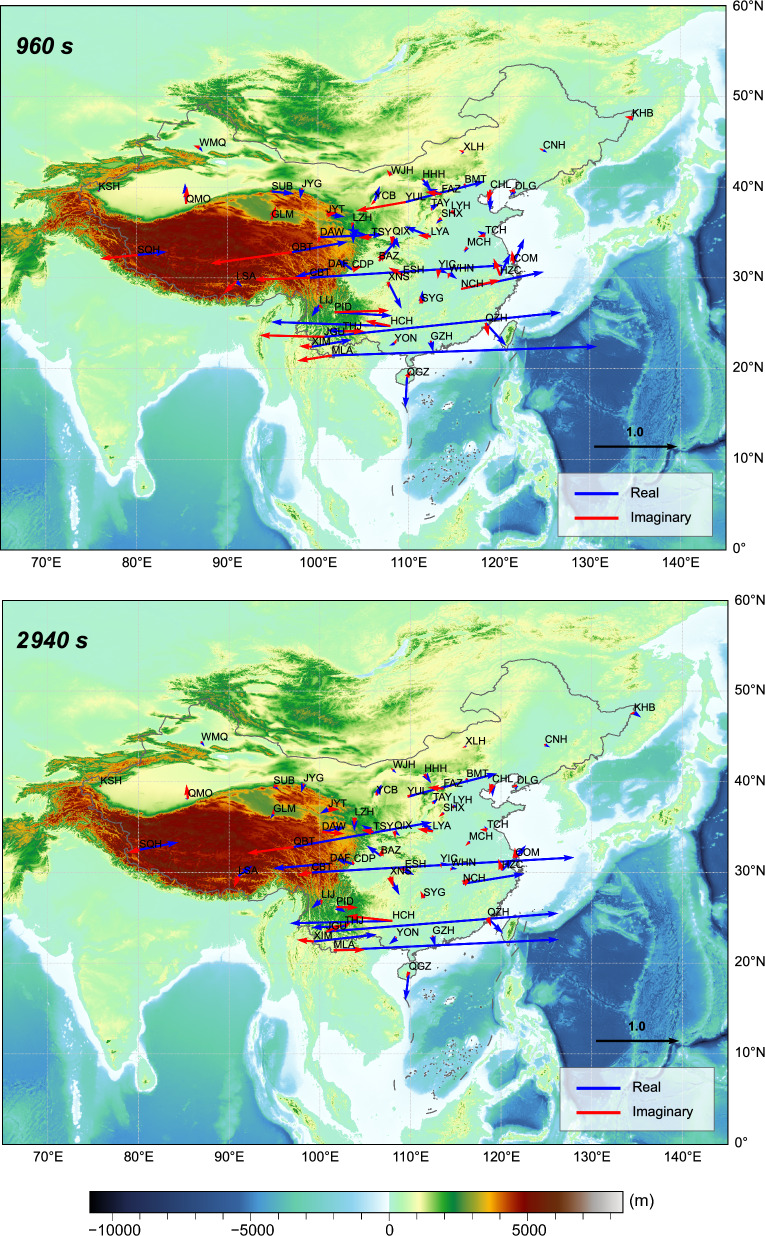
Tippers at two selected periods estimated at 54 observatories across China. Real parts of induction arrows point to conductors

**Fig. 3:**
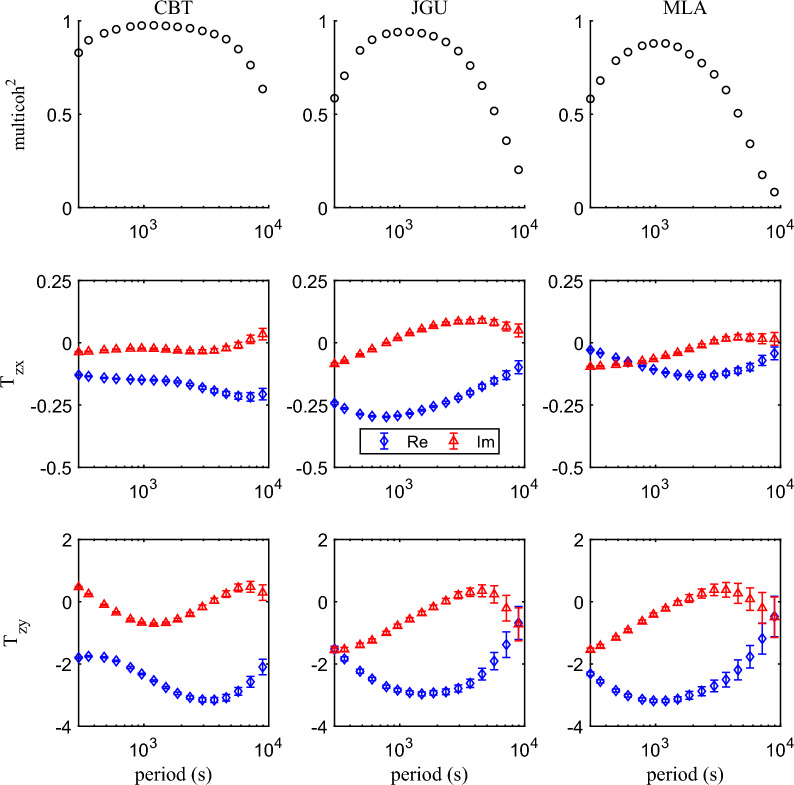
Coherency, tippers, and their uncertainties estimated at CBT (left), JGU (middle), and MLA (right) observatories

Figure 2 shows the maps of tippers in the form of induction arrows at periods 960 and 2940 s. As expected, the real part of induction arrows at coastal and island observatories point to the nearest deepest ocean (not very deep, however, thus giving relatively small tippers). What was completely unexpected was observing the abnormally large induction arrows at three inland (CBT, JGU, and MLA) observatories in southwest China. Real parts of induction arrows at these observatories reach values of around 3 and point predominantly to the east, thus implying substantially anomalous current concentration in the north–south direction to the east of the observatories’ locations. Figure 3 presents squared coherencies, tippers, and their uncertainties —as functions of the period—estimated at the discussed observatories. High coherency ($> 0.6$), smooth tippers’ behavior (with respect to periods), and their small uncertainties demonstrate the credibility of the tippers’ estimates at all observatories at periods as high as 5000 s. At larger periods, the coherency significantly deteriorates at JGU and MLA observatories leading to larger uncertainties in corresponding tippers’ estimates: this is most probably due to the degraded signal/noise ratio at those observation sites.

We also notice that being rather similar, the tipper’s behavior slightly varies with the observatory. The real part of $T_{zy}$ at CBT observatory reaches maximum magnitude (somewhat larger than 3) at a period of 3000 s, whereas at observatories JGU and MLA, maximum magnitudes (somewhat smaller than 3) are observed at periods 1500 and 1000 s, respectively. At roughly the same periods, the reversal of the imaginary parts occurs, which is in agreement with the results of Marcuello et al. (2005). Noteworthy, imaginary parts of $T_{zy}$ are three times smaller compared with corresponding real parts. As for real and imaginary parts of $T_{zx}$, they are more than one order of magnitude smaller than those of $T_{zy}$ at all three observatories, thus explaining the eastward direction of induction arrows (see Fig. 2).

**Fig. 4:**
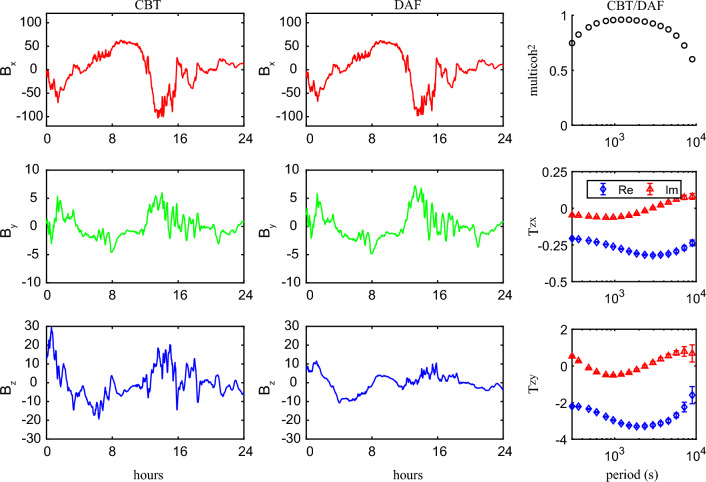
Two left panels: magnetic field time series at CBT and DAF observatories for geomagnetically disturbed day, September 7, 2017. Right panel: results of estimation of inter-site (CBT/DAF) tipper. See details in the text

To exclude the speculations that these extreme values of tippers are due to noise-related problems or/and issues in instruments’ installation (for instance, misalignment of the horizontal components or/and wrong calibration of one or other field component), we compared the time series of the magnetic field at CBT (where we observed the largest—among three observatories—tippers) and at DAF observatory (where we obtained moderate values of tippers). Two left plots in Fig. 4 present the comparison of CBT and DAF all components’ time series for the geomagnetically disturbed day, September 7, 2017. The disturbed day is chosen to make the comparisons more illustrative, but the same inferences (discussed below) are valid for any time interval. Even though CBT and DAF observatories are separated by $\sim 200 \,\hbox {km}$, it is seen that the shape and magnitude of horizontal component variations are very similar across all periods. This, in particular, means no issue exists in CBT horizontal field measurements. As for the vertical component, one can observe that the short-period CBT variations (those used to estimate tippers) have a much higher magnitude than corresponding DAF variations. The latter fact explains the immense value of tippers at CBT. Note that the longer-period (daily) variations are similar at both sites, thus excluding the speculation that the vertical component has a calibration issue. To put even more weight on the above results, we estimated inter-site tippers, considering in the right-hand side of Eq. (1) the horizontal components of DAF (instead of CBT). The right plot in Fig. 4 presents the results of estimation in the same manner as it was done in Fig. 3. As we expected, the single-site (CBT) and inter-site (CBT/DAF) tippers agree very well, thus confirming again that the CBT horizontal components are trustworthy. Similar comparisons and estimation of inter-site tippers performed for JGU and MLA observatories (not shown in the paper) also revealed no anomalies in the JGU and MLA data.

## Geological background

**Fig. 5:**
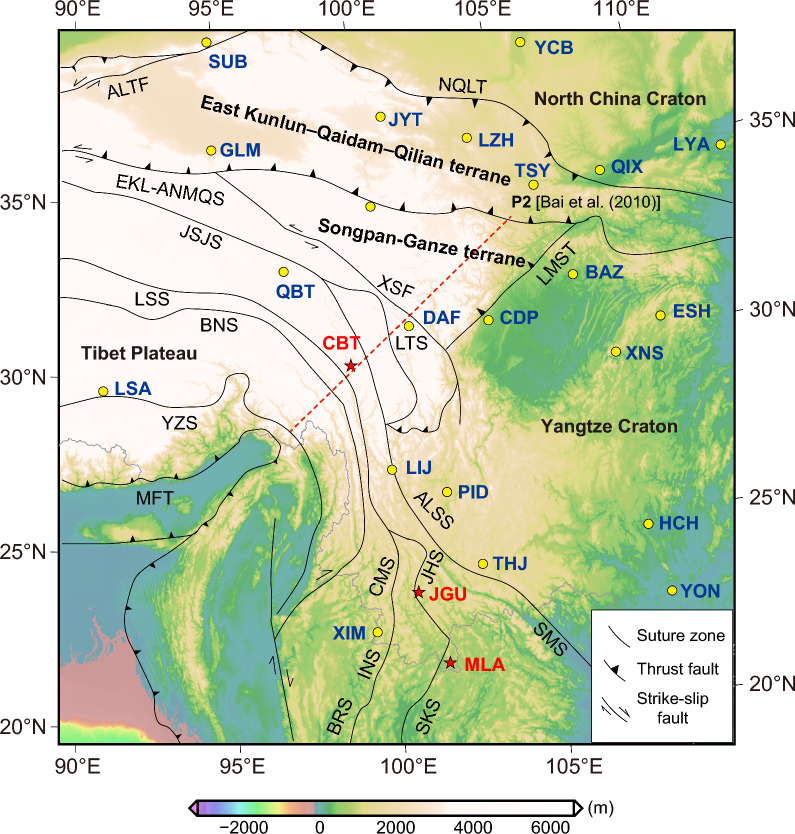
Simplified tectonic map (modified from Xu et al. (2015)) and locations of the three observatories (CBT, JGU, and MLA) with enormously large tipper values in southwest China. Red dashed line denotes the MT profile P2 in Bai et al. (2010). Suture zones and faults: ALTF: Altyn Tagh Fault; NQLT: North Qilian Thrust; EKL-ANMQS: East Kunlun–A’nyemaqen Suture; JSJS: Jinshajiang Suture; ALSS: Ailaoshan Suture; SMS: Song Ma Suture; LTS: Litang Suture; LMST: Longmenshan Thrust; LSS: Longmu Tso–Shuanghu Suture; BNS: Bangonghu–Nujiang Suture; YZS: Yarlung–Zangbo Suture; MFT: Main Frontal Thrust; CMS: Changning–Menglian Suture; INS: Inthanon Suture; BRS: Bentong Raub Suture; JHS: Jinghong Suture; SKS: Sra Kaeo Suture; XSF: Xianshuihe fault. The Yangtze craton is separated from the Indochina Block by the Ailaoshan–Songma suture

The three observatories with enormously large tippers are located in the area created by the multi-time amalgamation and collision of the Indochina Block with Tibet and the South China Block (Fig. 5). The observatories are located west of the Jinshajiang–Ailaoshan–Songma suture, which is the boundary between the Indochina Block and the Yangtze craton. The complex suture and fault system developed as the Paleo-Tethyan oceanic realm diminished. The Jinshajiang–Ailaoshan suture zone occurs between the Songpan–Ganze, the North Qiangtang–Qamdo–Simao-Indochina terrane, and the South China block and curves southward to connect with the Song Ma suture zone (Lepvrier et al. 2008). It is inferred that the Jinshajiang–Ailaoshan suture zone marks a closed branch of the main Paleo-Tethyan Ocean. Regional studies show that the Jinshajiang–Ailaoshan-Song Ma Paleo-Tethyan oceanic slab was subducted westward beneath the North Qiangtang–Simao-Indochina terrane along the Jinshajiang–Ailaoshan trenches. The Song Ma suture zone has been considered by many scientists as the boundary between the South China and Indochina blocks that was created by the closure of Paleo-Tethys ocean (Lepvrier et al. 2004). A detailed study of eclogite from the Song Ma suture zone has revealed the peak P–T conditions of $700\,^{\circ }\hbox {C}$ and  2.6 GPa. The oceanic protolith of this eclogite appears to have subducted to a mantle depth of  85 km (Zhang et al. 2012). U-Pb SHRIMP dating of zircon from this eclogite has yielded an age of 230 Ma, which constrains the timing of westward subduction and the closure of a Paleo-Tethyan oceanic basin between the South China and Indochina blocks as the Carnian (early–late Triassic). A previous magnetotelluric (MT) survey in the region, along the profile depicted as a red-dashed line in Fig. 5, images two channels of high electrical conductivity (1 S/m) at a depth of 20–40 km, which extend more than 800 km from the Tibetan plateau horizontally into southwest China (Bai et al. 2010). Note that Bai et al. (2010) did not present tipper data in their study.

Considering the geological background and the results of Bai et al. (2010), we design a conceptual 3-D conductivity model of the region and perform 3-D EM simulations to find modeling parameters that result in tippers comparable in magnitude and behavior with those observed.

## 3-D EM model study

### Designing 3-D conductivity model

In building the 3-D model, we additionally exploit the following two facts. First, we notice that the most north (CBT) and the most south (MLA) observatories are spaced apart for around 800 km; however, tippers at CBT and MLA show similar behavior and have comparable maximum magnitudes. This fact suggests that the zone of anomalous current concentration extends more than 800 km. Second, much smaller tippers observed at the neighboring (CDP) observatory (located around 200 km to the east-northeast of the CBT observatory) indicates that the anomalous zone’s width is likely less than 200 km.

**Fig. 6:**
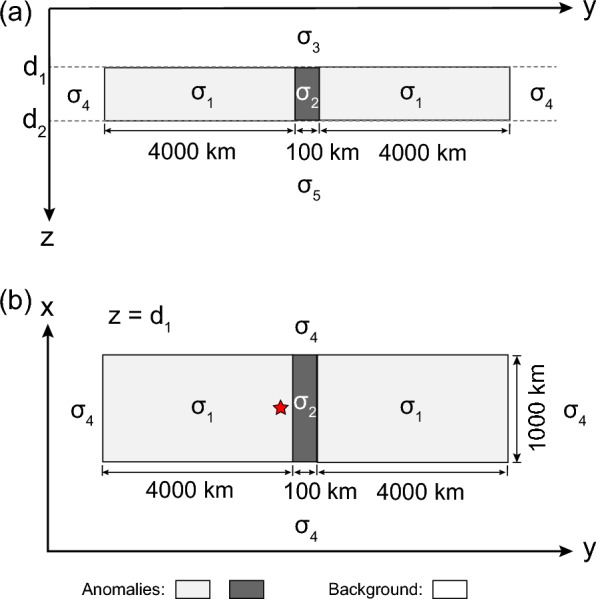
Illustration of the 3-D conductivity model used in the modelings. **a** Side view; **b** plan view. The red star denotes the location where we compare modeled tippers with observed tippers

With all the above information in mind, we constructed a 3-D conductivity model, which is shown in Fig. 6; the top and bottom plots in the figure, respectively, present side and plain views of the model. The model comprises two $l_x = 1000$ km $\times$ $l_y= 4000$ km resistive blocks of $\sigma _1$ separated by $w_y = 100$ km wide conductive zone of $\sigma _2$. This model setup allowed us to mimic the amalgamation of two resistive continental structures along a conductive suture zone. The conductivities $\sigma _1$, and $\sigma _2$ as well as the depths to the top, $d_1$, and to the bottom, $d_2$, of the layer containing the elongated conductor and massive resistive blocks varied during modeling. This 3-D part of the model was embedded in three-layered host media of respective conductivities $\sigma _3$, $\sigma _4$, and $\sigma _5$, which are also varied during simulations. Asterisk at the bottom plot depicts a location where the modeled tippers have the largest amplitudes; we took modeled tippers at this location when comparing modeled and observed results. To calculate tippers, we exploited a forward modeling tool, PGIEM2G (Kruglyakov and Kuvshinov 2018), which is based on a volume integral equation (IE) approach with IE contracting kernel (Pankratov and Kuvshinov 2016). Since we worked with the IE-based forward problem engine, the modeling domain was confined to the 3-D part of the model; thus, its size is given as $l_x \times (2l_y + w_y) \times h_z = 1000 \times 8100 \times h_z$ $\hbox {km}^3$, where $h_z =d_2 - d_1$ is the thickness of the layer comprising the 3-D anomalies. Laterally, the modeling domain was discretized by a uniform grid with cell sizes of 10 km in both *x* and *y* directions. The number of cells in the vertical direction depended on $h_z$ but did not exceed 4, due to the relatively small thickness of the layer comprising anomalies. The behavior of the EM field within (volume) cells was approximated by the first-order polynomials in all directions. Note that we performed extensive model experiments to justify the selected cell sizes and order of the polynomials.

### Results and discussion

We pursued a trial-and-error approach to find the models in which tippers behave as those observed and have comparable amplitudes. As mentioned above, in the course of simulations, we varied most of the parameters describing the model, excluding the lateral sizes of the resistive blocks and conducting zone, which were chosen according to the considerations discussed earlier in the paper.

General (somewhat anticipated) observations from the performed numerical experiments are as follows. The tippers become larger provided: (a) the elongated conductor (sandwiched between resistive blocks) is set closer to the surface; (b) the lateral conductivity contrast between the conductor and resistive blocks enlarges; and (c) the layers above and below the 3-D part of the model get less conductive. The results presented from now on are for the following model parameters: $\sigma _1 = \sigma _3 = \sigma _5 = 10^{-4}$ S/m and $\sigma _2 = 1$ S/m; these parameters were chosen to secure the largest values of tippers.

**Table 1:** Details of the 3-D models, results from which are discussed in the paper

Model	$\text{ d}_1[\text {km}]$	$\text{ d}_2\,[\text {km}]$	$\sigma _4 \,[\text {S/m}]$	$\max |\mathop {\textrm{Re}} T_{zy} |$
M1	5	15	$10^{-4}$	0.33
M2	5	15	$10^{-1}$	2.89
M3	5	15	1	3.69
M4	20	40	1	2.54

The last column also presents the maximum modeled amplitudes of the real part of $T_{zy}$

**Fig. 7:**
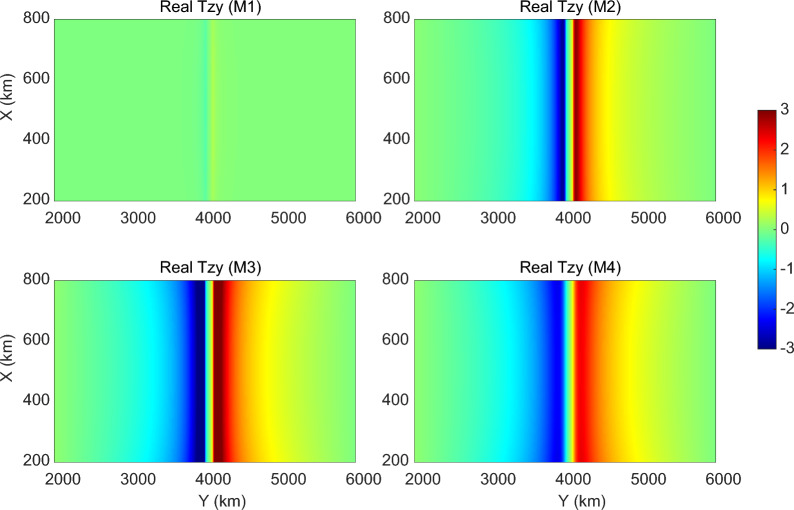
Maps of modeled real parts of $T_{zy}$ component at a period of 2940 s. See details in the text

**Fig. 8:**
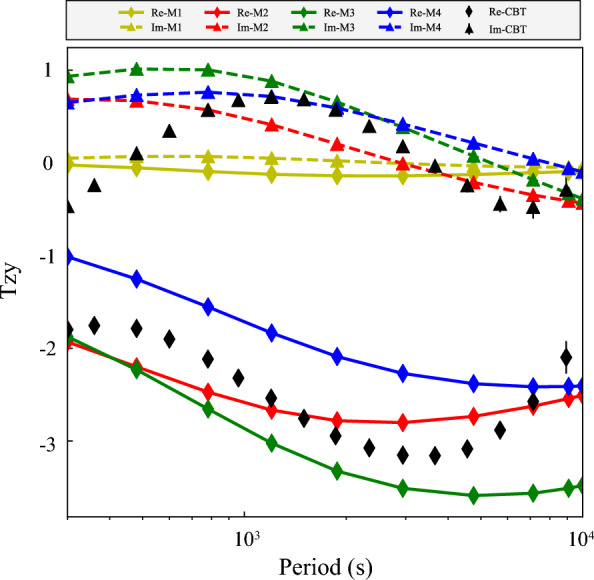
Modeled real and imaginary parts of $T_{zy}$ at the location where maximum modeled amplitudes are obtained. For comparison, the experimental tippers at CBT are also plotted. See details in the text

We first considered the 3-D conductivity model (depicted as M1 in Table 1 and in Figs. 7 and 8) in which the elongated conductor abuts the high resistive environment, meaning that $\sigma _4$ is taken as $10^{-4}$ S/m. As seen from the table and figures, the tippers in this model are far below the observed values. Thus, just a presence in the model of the elongated strong conductor surrounded by much resistive medium cannot explain extremely large observed tippers. Hereinafter, we show and discuss only $T_{zy}$ component of tippers because modeled and observed $T_{zx}$ components are negligibly small, compared to $T_{zy}$ component. Moreover—in order not to overload the presentation—we show only the real part of $T_{zy}$, noticing that the imaginary part is smaller than the real part, both in modeled and observed tippers (see Fig. 3). Note that Fig. 7 presents the spatial distribution of real parts of modeled $T_{zy}$ at a period of 2940 s (at which maximum amplitudes of the experimental tippers are detected), whereas in Fig. 8 the real parts of $T_{zy}$ are shown as a function of the period at location (depicted as an asterisk in the bottom plot of Fig. 6) where the largest modeled tippers are obtained. Remarkably, changing the value of $\sigma _4$ from $10^{-4}$ S/m to higher values ($10^{-1}$ S/m in model M2 and 1 S/m in models M3 and M4) dramatically enlarges amplitudes of tippers (see, again, Table 1 and Figs. 7 and 8) making them compatible with the observed amplitudes. Such tippers’ substantial enhancement becomes possible due to the so-called current channeling effect (Jones 1983), which manifests as an excessive current concentration in the elongated conductor. Indeed, in models M2–M4, the current can flow in and out of the elongated conductor; the situation was not possible in model M1 where the current appeared locked in the conductor. It is worth mentioning that in models M1–M3, the conductor has a thickness of 10 km, and its top boundary is placed at a depth of 5 km.

Figure 7 presents the results from M1–M4 models in the central $600 \times 4000 \times 4000$ $\hbox {km}^3$ part of the modeling region, illustrating several facts, namely: a) tippers’ smallness in the M1 model; b) symmetry of the results (up to the sign in *y*-direction) with respect to the location of the elongated conductor; c) different patterns of tippers’ decay in *y*-direction with respect to the model parameters; d) weak variations of tippers in *x* direction. The latter is due to the conductor extension, asserting that the modeled results shown in Fig. 8 for a location depicted by an asterisk in the bottom plot in Fig. 6 are representative and reflect the general behavior of the modeled tippers with respect to the period. Note that in Fig. 8, along with modeled results, we show tippers estimated from the data (black diamonds and triangles). In order not to overburden the plot, we demonstrate experimental tippers estimated at the CBT observatory where we observed maximum (among those estimated at three considered observatories; see bottom plots in Fig. 3) tippers’ amplitudes.

As is seen, the modeled tippers are the largest in the M3 model, i.e. one with $\sigma _4 = 1$ S/m (see Figs. 7 and 8) and they even exceed the observed tippers. Placing the conductor deeper (at a depth of 20 km, in an attempt to mimic the conductor revealed by Bai et al. (2010)) and increasing its thickness to 20 km (in an attempt to compensate for the deeper location; M4 model) makes, however, modeled tippers smaller than those observed. Notably, maximum amplitudes of tippers in the M3 and M4 models (cf. respective green and blue curves in Fig. 8) are observed at much longer periods compared to CBT results. When the conductivity contrast between the channel and its background increases to $10$ (as in model M2, red curves) maximum amplitude of the real part of $T_{zy}$ becomes closest to the CBT results, and, moreover, maximum is seen at a comparable period. Summing up, our model study suggests that enormously large tippers can be reproduced when the study area is subjected to current channeling. The final comment of this section is that it could be understood that our model experiment is not exhaustive in terms of full exploration of model parameter space, but the experiment has a goal to demonstrate that the observed huge tippers can be explained by the 3-D models mimicking the tectonic/geological setting of the region.

## Conclusion and outlook

In this study, we processed 12 years of continuous minute-mean magnetic field time series obtained at a continental net of 54 geomagnetic observatories across China in order to estimate vertical transfer functions—tippers—in as wide a period range as feasible. Enormously large tipper amplitudes reaching the value of 3 were observed at three inland observatories in southwest China; as far as we know, such large tippers were not documented in any region of the world.

We designed 3-D conductivity models that mimic the tectonic/geological setting of southwest China and performed simulations aiming to reproduce enigmatically huge tippers in the region. Our model study suggests that such large tippers can be attributed to the current channeling. We acknowledge that tippers in this area can be produced by a more complex suture and fault system of different geometries (e.g., offset, strike, dipping direction). Therefore, further 3-D inversion of tippers is required and will be performed in the future to distinguish the nature of the conductive channel, either caused by ancient sutures or subduction zones in southwest China.

## Data Availability

The modeling tool used in this research is available at https://gitlab.com/m.kruglyakov/PGIEM2G. The observed tipper data and modeling results are archived at https://doi.org/10.5281/zenodo.7734623.

## Abbreviations

EM: Electromagnetic
MT: Magnetotelluric
2-D: Two-dimensional
3-D: Three-dimensional
IE: Integral equation

## References

[CR1] Bai D, Unsworth MJ, Meju MA, Ma X, Teng J, Kong X, Sun Y, Sun J, Wang L, Jiang C (2010). Crustal deformation of the eastern Tibetan plateau revealed by magnetotelluric imaging. Nat Geosci.

[CR2] Berdichevsky MN, Dmitriev VI (2008). Models and methods of magnetotellurics.

[CR3] Chamalaun F, Barton C (1993). Electromagnetic induction in the Australian crust: results from the Australia-wide array of geomagnetic stations. Explor Geophys.

[CR4] Chen C, Kuvshinov A, Kruglykov M, Munch F, Rigaud R (2023). Constraining the crustal and mantle conductivity structures beneath islands by a joint inversion of multi-source magnetic transfer functions. J Geophys Res Solid Earth.

[CR5] Jones AG (1983). The problem of current channelling: a critical review. Geophys Surv.

[CR6] Karato S-I, Wang D (2013). Electrical conductivity of minerals and rocks. Phys Chem Deep Earth.

[CR7] Kruglyakov M, Kuvshinov A (2018). Using high-order polynomial basis in 3-D EM forward modeling based on volume integral equation method. Geophys J Int.

[CR8] Kruglyakov M, Kuvshinov A (2022). Modelling tippers on a sphere. Geophys J Int.

[CR9] Lepvrier C, Maluski H, Van Tich V, Leyreloup A, Thi PT, Van Vuong N (2004). The early Triassic Indosinian orogeny in Vietnam (Truong Son Belt and Kontum Massif); implications for the geodynamic evolution of Indochina. Tectonophysics.

[CR10] Lepvrier C, Van Vuong N, Maluski H, Thi PT, Van Vu T (2008). Indosinian tectonics in Vietnam. Comptes Rendus Geosci.

[CR11] Macmillan S, Olsen N (2013). Observatory data and the swarm mission. Earth Planets Space.

[CR12] Marcuello A, Queralt P, Ledo J (2005). Applications of dispersion relations to the geomagnetic transfer function. Phys Earth Planet Inter.

[CR13] Pankratov O, Kuvshinov A (2016). Applied mathematics in EM studies with special emphasis on an uncertainty quantification and 3-D integral equation modelling. Surv Geophys.

[CR14] Parkinson WD, Jones FW (1979). The geomagnetic coast effect. Rev Geophys.

[CR15] Püthe C, Kuvshinov A (2014). Mapping 3-d mantle electrical conductivity from space: a new 3-d inversion scheme based on analysis of matrix q-responses. Geophys J Int.

[CR16] Rigaud R, Kruglyakov M, Kuvshinov A, Pinheiro K, Petereit J, Matzka J, Marshalko E (2021). Exploring effects in tippers at island geomagnetic observatories due to realistic depth- and time-varying oceanic electrical conductivity. Earth Planet Space.

[CR17] Thiel S, Heinson G, Gray DR, Gregory RT (2009). Ophiolite emplacement in NE Oman: constraints from magnetotelluric sounding. Geophys J Int.

[CR18] Wang L, Hitchman AP, Ogawa Y, Siripunvaraporn W, Ichiki M, Fuji-ta K (2014). A 3-D conductivity model of the Australian continent using observatory and magnetometer array data. Geophys J Int.

[CR19] Xu Z, Dilek Y, Cao H, Yang J, Robinson P, Ma C, Li H, Jolivet M, Roger F, Chen X (2015). Paleo-Tethyan evolution of Tibet as recorded in the East Cimmerides and West Cathaysides. J Asian Earth Sci.

[CR20] Yoshino T, Katsura T (2013). Electrical conductivity of mantle minerals: role of water in conductivity anomalies. Annu Rev Earth Planet Sci.

[CR21] Zhang K-J, Zhang Y-X, Tang X-C, Xia B (2012). Late Mesozoic tectonic evolution and growth of the Tibetan plateau prior to the Indo-Asian collision. Earth Sci Rev.

